# AZD2014 Radiosensitizes Oral Squamous Cell Carcinoma by Inhibiting AKT/mTOR Axis and Inducing G1/G2/M Cell Cycle Arrest

**DOI:** 10.1371/journal.pone.0151942

**Published:** 2016-03-31

**Authors:** Chih-Chia Yu, Hsien-bin Huang, Shih-Kai Hung, Hui-Fen Liao, Ching-Chih Lee, Hon-Yi Lin, Szu-Chin Li, Hsu-Chueh Ho, Chung-Lin Hung, Yu-Chieh Su

**Affiliations:** 1 Department of Life Science and Institute of Molecular Biology, National Chung Cheng University, Chia-Yi, Taiwan, R.O.C; 2 Division of Hematology-Oncology, Department of Internal Medicine, Buddhist Dalin Tzu Chi Hospital, Chia-Yi, Taiwan, R.O.C; 3 School of Medicine, Tzu Chi University, Hualian, Taiwan, R.O.C; 4 Department of Radiation Oncology, Buddhist Dalin Tzu Chi Hospital, Chia-Yi, Taiwan, R.O.C; 5 Department of Otolaryngology, Buddhist Dalin Tzu Chi Hospital, Chia-Yi, Taiwan, R.O.C; 6 Department of Biochemical Science and Technology, National Chiayi University, Chia-Yi, Taiwan, R.O.C; National Health Research Institutes, TAIWAN

## Abstract

**Background:**

Oral squamous cell carcinoma (OSCC) is one of the most common malignant neoplasms in Taiwan. Activation of the mTOR signaling pathway has been linked to decreased radiation responsiveness in human oral cancer, thus it limits efficacy of radiotherapy. To address this question, we investigated the effect of AZD2014, a novel small molecular ATP-competitive inhibitor of mTORC1 and mTORC2 kinase, as a radiosensitizer in primary OSCC and OSCC-derived cell line models.

**Methods:**

We isolated primary tumor cells from OSCC tissues and cell lines. AZD2014 was administered with and without ionizing radiation. The radiosensitizing effect of AZD2014 were then assessed using cell viability assays, clonogenic survival assays, and cell cycle analyses. Western blotting was used to detect protein expression.

**Results:**

Combination treatment with AZD2014 and irradiation resulted in significant reduction in OSCC cell line and primary OSCC cell colony formation due to the enhanced inhibition of AKT and both mTORC1 and mTORC2 activity. Pre-treatment with AZD2014 in irradiated oral cancer cells induced tumor cell cycle arrest at the G1 and G2/M phases, which led to disruption of cyclin D1-CDK4 and cyclin B1-CDC2 complexes. Moreover, AZD2014 synergized with radiation to promote both apoptosis and autophagy by increasing caspase-3 and LC3 in primary OSCC cells.

**Conclusions:**

These findings suggest that in irradiated OSCC cells, co-treatment with AZD2014, which targets mTORC1 and mTORC2 blockade, is an effective radiosensitizing strategy for oral squamous cell carcinoma.

## Introduction

In Taiwan, oral cancer is the fourth most frequent cause of death from cancer among males [1]. Radiation therapy (RT) is often used to treat oral cancer; however, outcomes for RT are unsatisfactory due to the high risk of regional or distant metastases and local failure. Therefore, the development of strategies for improving sensitivity to RT is required.

The mammalian target of rapamycin (mTOR) is a key regulator of translation that controls cell growth, proliferation, survival, and angiogenesis, and which is frequently dysregulated in tumor cells [2]. Two distinct mTOR signaling complexes have been identified: mTORC1 (mTOR-raptor) and mTORC2 (mTOR-rictor). The 70-kDa ribosomal protein S6 kinase 1 (p70S6K1) and eukaryotic translation initiation factor 4E-binding protein 1/eukaryotic translation initiation factor 4E (4EBP1/eIF4E), two major downstream effectors of mTORC1, play important roles in multiple cellular functions and aberrant activation of signaling that leads to cancer transition. In addition, mTORC2 phosphorylates AKT at Ser473, affecting AKT-mediated survival signaling and thereby modulating cell motility [3]. mTOR inhibitors, which have been utilized in clinical trials as targeted therapies, show greater therapeutic benefits when combined with other treatments [4]. The mTOR inhibitors can potentially be used as single therapeutic agents, or in combination with RT or chemotherapeutic agents, to obtain synergistic repression of oral cancer [5]. However, most studies that targeted the mTOR pathway in cancer therapy have focused on allosteric mTOR inhibitors. Allosteric mTOR inhibitors, which inhibit mTORC1 but not mTORC2 [6,7], result in feedback activation of AKT signaling, which can attenuate their antitumor activity [8–10]. Previously, we have also shown that the mTORC1-specific inhibitor, RAD001, enhanced radiosensitization in SCC4 oral cancer cells. However, due to AKT signaling induced via feedback activation, an effect for RAD001 on reducing p-4EBP1 levels was absent or weak. This finding may indicate a limited effectiveness of mTORC1-targeting therapies for suppressing tumor activity [11].

AZD2014 is a newer, second generation mTOR inhibitor that blocks activation of both mTORC1 (phosphorylation of 70S6K1 and 4EBP1) and mTORC2-mediated AKT Ser473 phosphorylation, and activates apoptosis in cancer cells [9]. Moreover, AZD2014 has been shown to increase radiosensitivity in glioblastoma stem-like cells (GSCs) [12]. Thus, AZD2014 may be a better therapeutic agent than mTORC1 inhibitors to enhance the antitumor activity of radiation in oral squamous cell carcinoma (OSCC). Due to the fact that cell lines cannot represent the diversity of human cancers from patient tumors, we established primary oral cancer cell cultures from tissues of oral cancer patients and used OSCC cell lines as experimental models to explore the underlying mechanism of AZD2014-mediated radiosensitization. Our studies clearly demonstrate that the combined use of AZD2014 with RT results in significant synergy in suppressing OSCC cell growth. Thus, dual mTORC1/mTORC2 blockade is an effective radiosensitizing strategy against OSCC cells.

## Materials and Methods

### Reagents and chemicals

AZD2014 was obtained from AstraZeneca (London, United Kingdom), dissolved in DMSO at a concentration of 10 mM, and stored at −20°C until further use. The stock solution was diluted to the appropriate concentration in culture medium containing serum just before addition to cell cultures. All antibodies used in this work were obtained from Cell Signaling Technology (Beverly, MA, USA).

### Tissue specimens and initial cell culture

Tumor tissues originated from the lip, buccal mucosa, and tongue of 3 patients with OSCC (61 to 70 years of age with newly diagnosed with either stage III or IVA). The primary specimens were collected surgically. This study was approved by the human research ethics committee of the Buddhist Dalin Tzuchi General Hospital (B10302008). All samples were obtained from consenting study subjects undergoing surgical tumor resection who signed a written informed consent approved by a human research ethics committee (B10302008). The tissues were washed three times in phosphate-buffered saline (PBS) containing 1% penicillin-streptomycin (10,000 U/ml penicillin and 10 mg/ml streptomycin). For cell dissociation, sample were minced into 1-to 2 mm pieces, then placed into digestion TrypLE^®^ Express Enzyme (Gibco Invitrogen, Carlsbad, CA) at 37° for 1 hour. The cells were mechanically broken apart by pipetting every 15 minutes during digestion. The samples were passed through a 100 μM nylon cell strainer and the cells were counted. The cells were centrifuged at 1100 rpm for 5 minutes, and then resuspended in keratinocyte growth media (KGM, ScienCell Research Laboratories, Carlsbad, CA) with 15% fetal bovine serum (FBS). Adherent primary cells were cultured to allow cell counting and other experiments.

### Cell lines and cell culture

The OSCC-derived cell lines SCC4 and SCC25, were purchased from the American Type Culture Collection (Manassas, VA, USA), cultured in DMEM/F12 containing 10% fetal bovine serum (FBS), 1% penicillin-streptomycin (10,000 U/ml penicillin and 10 mg/ml streptomycin), and 2 mM glutamine in 10 cm dishes at 37°C in a humidified atmosphere of 5% CO_2_ and 95% air.

### MTT assay

As previously described [11], cells (5×10^3^) were seeded in 96-well plates, cultured overnight, and then treated with either DMSO (vehicle) or AZD2014 (25–100 nM) for 48 hours. The cell viability after indicated treatment was measured by the 3-[4,5-dimethylthiazol-2-yl]-2,5-diphenyltetrazolium bromide (MTT) assay.

### Clonogenic assay

Primary OSCC cells and OSCC-derived cell lines were seeded into 10-cm cell culture plates (70 × 10^2^ cells/plate) and grown overnight. The cells were pre-treated with indicated treatments or vehicle (control) and irradiated at 0, 2, 4, or 6 Gy. After 14 days, colonies (defined as groups of >50 cells) were stained with 0.05% crystal violet and counted. The surviving fractions of treatment groups were normalized to the surviving fractions of corresponding controls (non-irradiated), as previously described [11].

### Cell cycle profiling

The effects on the cell cycle in OSCC-derived cell lines and primary OSCC cells were assessed with the NucleoCounter NC-3000 (ChemoMetec, Allerød, Denmark), which is based on the analysis of DAPI-stained cells.

### Western blotting

After treatment, cells were harvested and lysed, and protein concentrations were measured using the Bio-Rad protein assay kit (Bio-Rad, Richmond, CA, USA). Fifty micrograms of protein from each sample was separated on an SDS-PAGE gel and transferred to a PVDF membrane (Millipore, Billerica, MA, USA). After blocking with non-fat dry milk for 1 hour, membranes were incubated with primary antibodies overnight at 4°C, followed by incubation with secondary antibodies for 1 hour. Reactive bands were visualized using a chemiluminescence (ECL) detection kit (Millipore).

### Statistical analysis

All data are presented as the mean ± standard deviation. Significance levels were calculated using Student’s t-test, and p-values of less than 0.05 were considered statistically significant.

## Results

### Expression of AKT and mTOR signaling in OSCC cell lines

**Fig 1:**
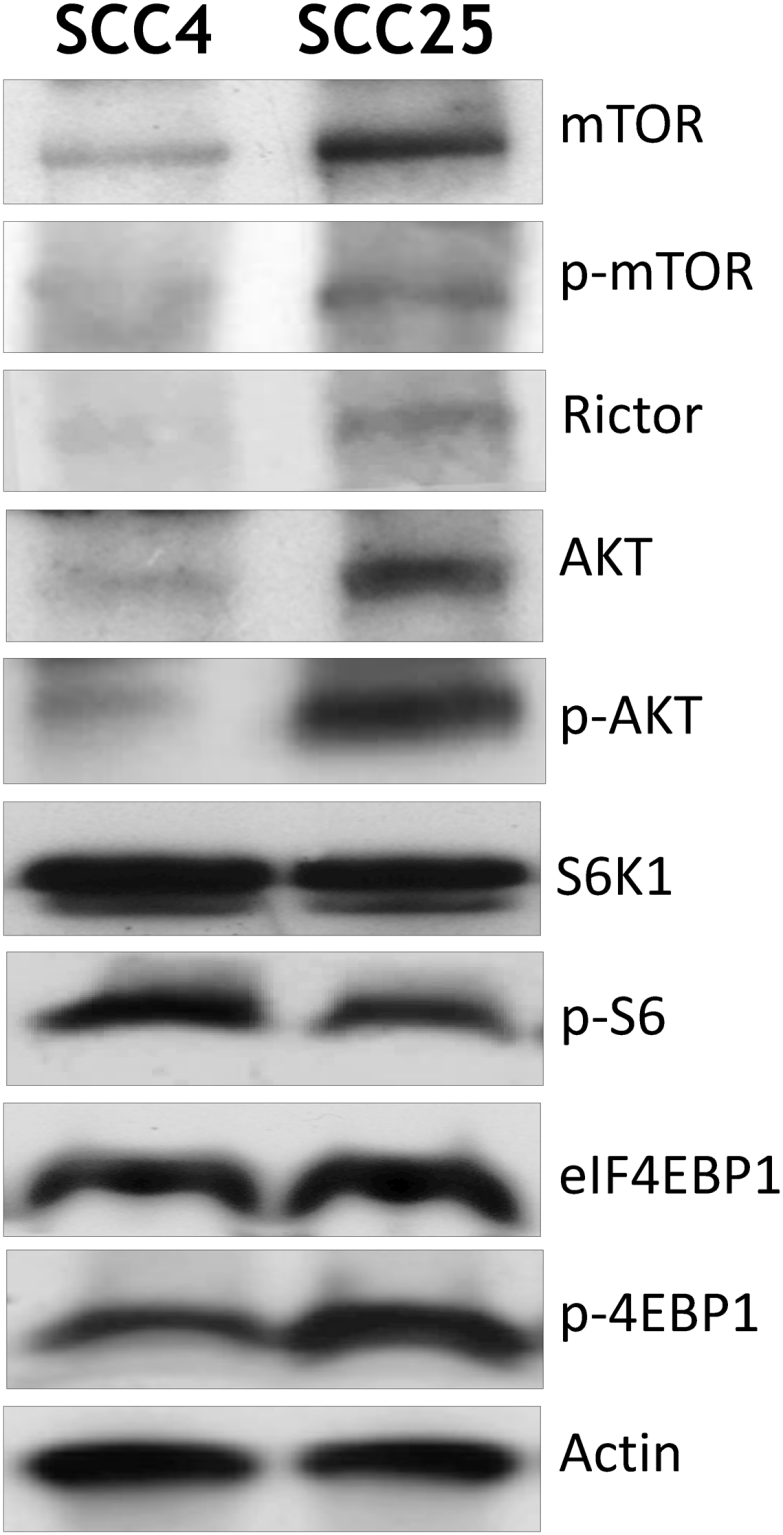
Expression of AKT and mTOR signaling pathway in OSCC cell lines. Cell lines were homogenized in lysate buffer as described in Materials and Methods. A 50 μM sample of protein was analyzed by Western blotting using specific antibodies against components of the AKT/mTOR pathway.

We first investigated AKT/mTOR signaling in OSCC-derived cell lines SCC4 and SCC25. Activation of mTOR can be reliably determined by phosphorylation of Ser2448, a site is that is phosphorylated by AKT. Activation of mTOR results in phosphorylation of two main downstream targets the ribosomal protein p70S6K and subsequent phosphorylation of the S6 ribosomal protein (p-S6). Another, eukaryotic translation initiation factor 4E binding protein1 (4EBP1) phosphorylated at Ser-65 corresponding to the hyperphosphorylated form, is regulated by mTORC1. Compared with the SCC4 cell line, activation of phospho-AKT at Ser473 was observed in SCC25 cells. High levels of phospho-mTOR at Ser2448 and phospho-4EBP1 at Ser65 were detected. Also phospho-S6 at Ser235/236 was not elevated in SCC25 cells. The mTORC2 adaptor protein, rictor displayed a significantly higher expression in the SCC25 cell line (Fig 1). The above results suggested the presence of highly activate mTOR signaling in OSCC cell lines.

### AZD2014 induces cytotoxicity in OSCC cell lines

**Fig 2:**
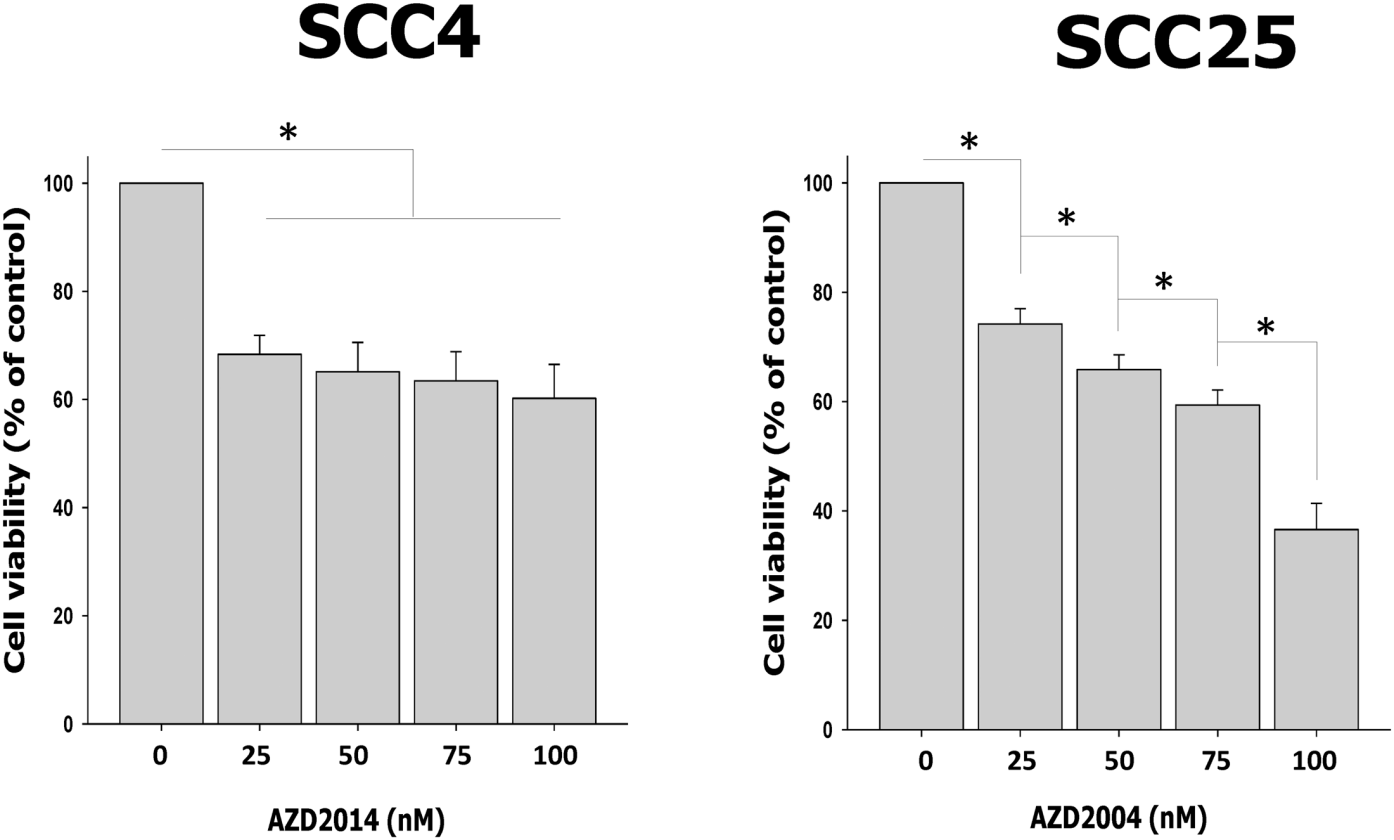
AZD2014 inhibited cell proliferation in OSCC cell lines. SCC4 and SCC25 cell proliferation after treatment with AZD2014 (0 to 100 nM) 48 hours using the MTT assay. Data are the mean ± SD of three independent experiments performed in triplicate. SD = standard deviation. *p<0.05 compared to the vehicle control.

To determine the effect of AZD2014 on cell survival, we treated OSCC cells with AZD2014 for 48 hours at concentrations ranging from 25 nM to 100 nM. SCC4 cells treated with AZD2014 at concentrations of 25, 50, 75 and 100 nM reduced cell viability by 31.64%, 34.86%, 36.56% and 39.79%, respectively. SCC25 cell viability was reduced by AZD2014 at concentrations of 25, 50, 75 and 100 nM by 25.83%, 34.14%, 40.63% and 63.40%, respectively after 48 hours. We found that AZD2014-treated SCC4 cells showed a maximum growth inhibition of about 40% even at a high concentration of 100 nM. However, SCC25 cells were more highly sensitive to AZD2014 (Fig 2).

### Combining AZD2014 with radiation significantly reduces colony formation in OSCC cell lines

**Fig 3:**
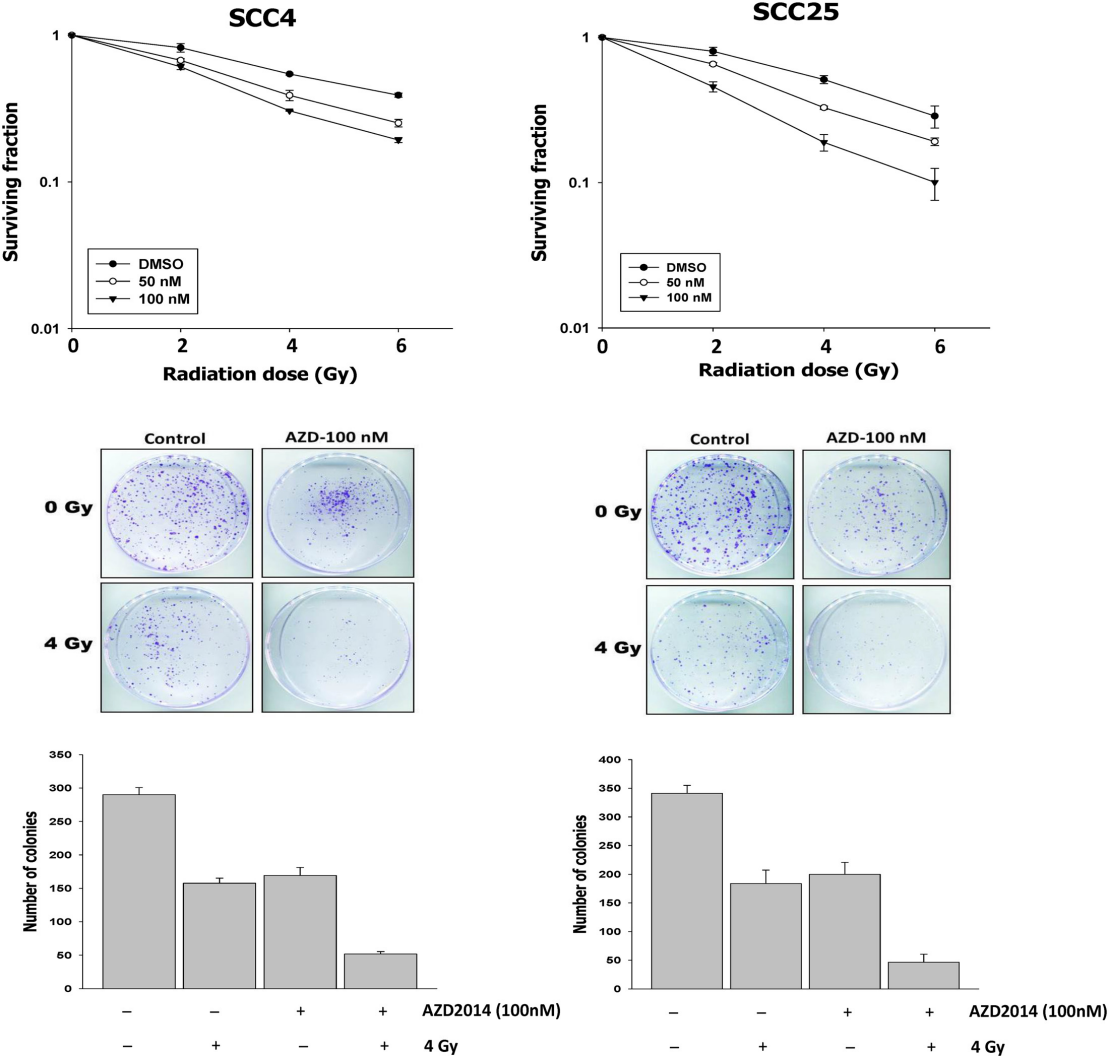
Effect of AZD2014 and radiation on SCC4 and SCC25 colony formation. (A) Cells were exposed to radiation (0–6 Gy) with and without AZD2014 (50 or 100 nM for 1 h) and cultured for 14 days. (B) The colonies were imaged at 14 days. The number of colonies in each well was counted. Data are the mean ± SD of three independent experiments performed in triplicate. SD = standard deviation.

To elucidate the effect of AZD2014 administration on the sensitivity of oral cancer cell lines to ionizing irradiation (IR), cells were pre-treated with either DMSO or AZD2014 (50 or 100 nM) for 14 days, followed by irradiation (0–6 Gy). Clonogenic assays demonstrated that combination treatment of AZD2014 and IR significantly reduced colony formation in SCC4 and SCC25 cells, compared with single inhibitor doses or IR alone (Fig 3). AZD2014 showed a dose-dependent enhancement of IR-induced inhibition of survival in both SCC4 and SCC25 cells.

### Combining AZD2014 with radiation inhibits AKT/mTOR signaling, but does not induce cell death pathways and γ-H2AX activity in OSCC cell lines

**Fig 4:**
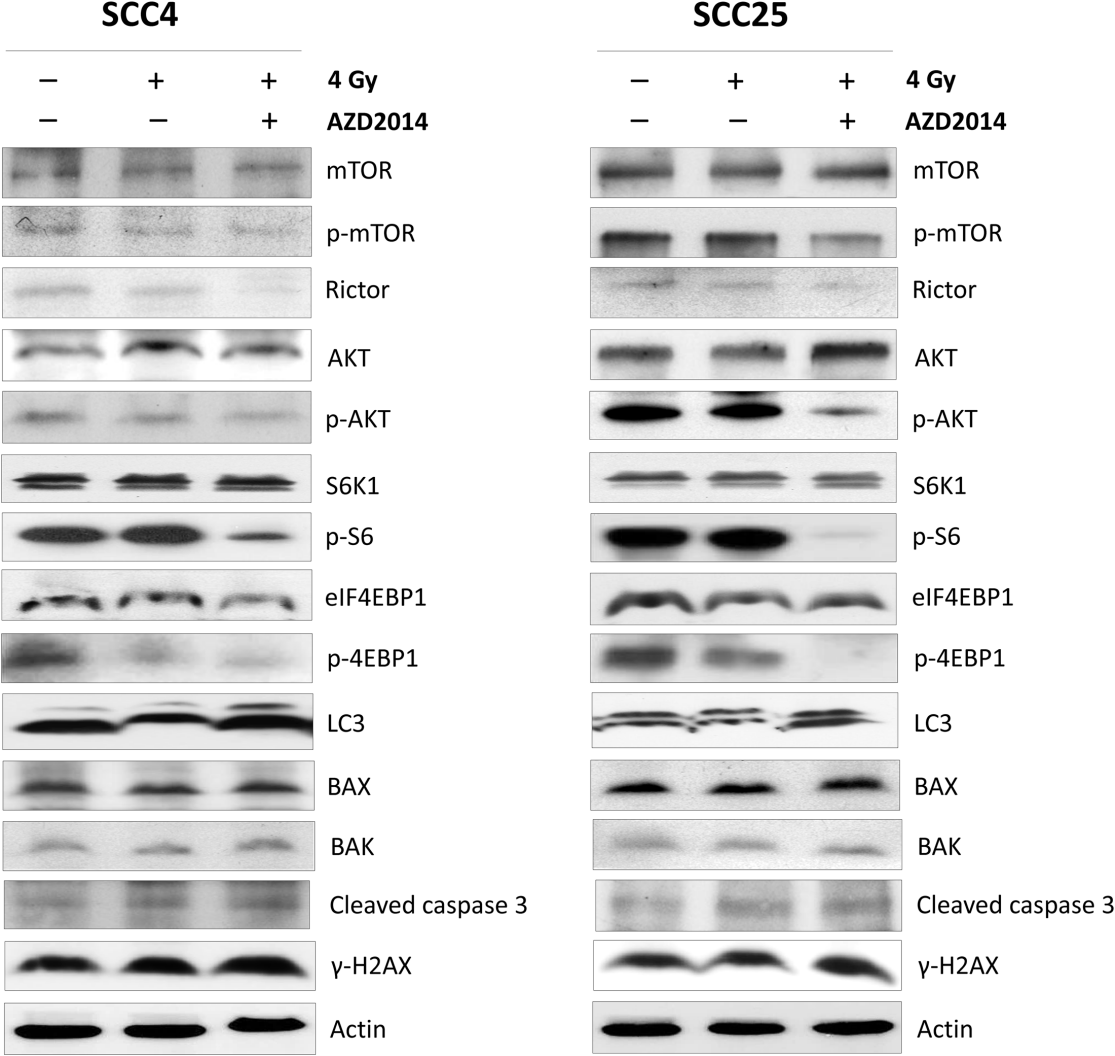
Effect of combination treatment with AZD2014 and IR or IR alone on AKT/mTOR pathway apoptosis, autophagy, and DNA damage pathway-related proteins in OSCC cell lines. SCC4 and SCC25 cells were treated with 100 nM AZD2014 with and without 4 Gy radiation. After 48 hours, the cells were harvested for preparation of whole-cell protein lysates and subsequent followed with Western blotting to detect the given proteins.

To understand the potential mechanisms of AZD2014-mediated radiosensitization, we first examined the effects of AZD2014 on activation of the AKT/mTOR pathway. In both SCC4 and SCC25 cells, treatment with AZD2014 and IR significantly down-regulated the levels of phospho-AKT, phospho-mTOR, the phosphorylation of S6 and 4EBP1, represents mTORC1 kinase activity whereas no significant changes were seen in IR alone. Likewise, we observed a significant decrease in protein levels of rictor, a component of mTORC2 protein, in SCC25 cells. Although the autophagy-related protein light chain 3 (LC3) expression levels increased in both cell lines after combination treatment with IR and AZD2014, the expressions of apoptosis-related proteins, such as BAX, BAK, were not significantly different between treatment and control groups. The cleaved caspase-3 expression was slightly higher in both cell lines after treatment with RT and combined with AZD2014. Moreover, the expression of γ-H2AX, a biomarker for DNA double-strand break,[13] showed no significant change with combination treatment (Fig 4). These findings indicate that AZD2014 may be effective as a radiosensitizing agent against OSCC cell growth, through enhanced inhibition of both AKT/mTORC1 and mTORC2 activity.

### Treatment with AZD2014 and radiation increases the percentage of cells in the G1 and G2/M phase of the cell cycle, and leading to altered levels of G1 and G2/M checkpoint regulators

**Fig 5:**
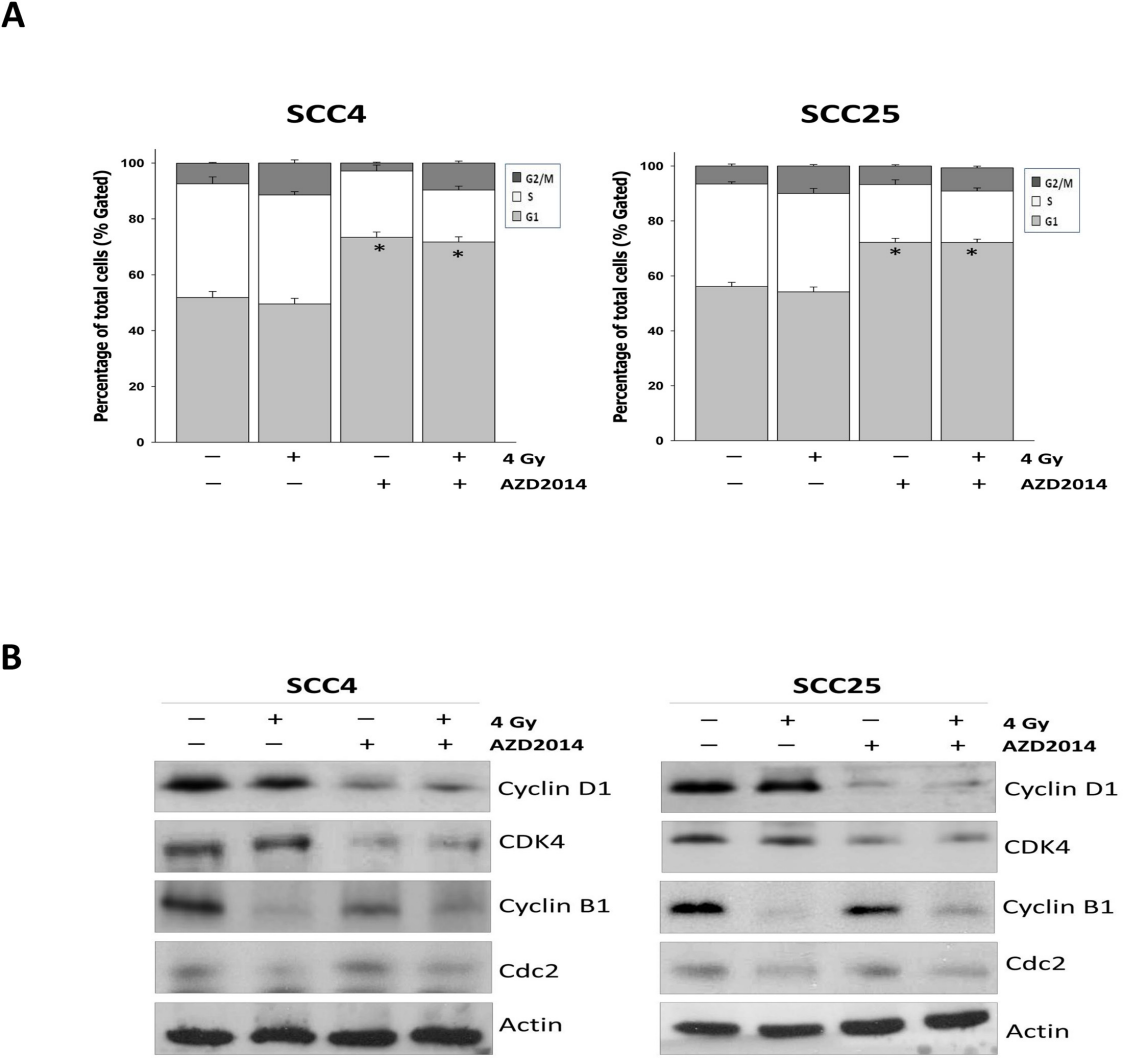
AZD2014 or IR alone and in combination treatment induced cell cycle arrest by altering the cell cycle checkpoints in OSCC cell lines. SCC4 and SCC25 cells were treated with AZD2014 alone, IR alone, and the two treatments in combination for 48 hours. Cell cycle profiles were determined using the NucleoCounter NC-3000. (A) Shows the percentage of cells in G1, S, and G2/M phases were quantified and results expressed at the mean ± SD of three independent experiments performed in triplicate. *p < 0.05 compared to the IR alone. (B) The cells were treated with AZD2014, IR alone, and in combination for 48 hours. Cell lysates were prepared and immunoblotted for cyclin D1, CDK4, cyclinB1 and CDC2 and actin as a loading control. SD = standard deviation.

The mTOR pathway positively controls cell cycle progression and cell proliferation by regulating S6K1 and the eukaryotic translation initiation factor, 4EBP1[14]. As radiosensitization by inhibition of mTOR signaling has been shown to cause cell cycle arrest [11,12], we assessed AZD2014-induced radiosensitization in SCC4 and SCC25 cells. A higher proportion of the cell population was detected in the G1 phase after treatment with AZD2014 as compared with radiation alone. Otherwise, we found that the proportion of G2/M phase cells increased in IR-treated cells. SCC4 and SCC25 cells pre-treated with AZD2014 and then irradiated showed significantly increased proportions at the G1 stage and a G2/M delay (Fig 5A). To understand the mechanisms by which AZD2014 combined with radiation induces G1 and G2/M phase arrest to inhibit cell growth, we tested the likelihood of changes in expression levels of diverse regulators of the cell cycle particularly associated with G1 checkpoints such as cyclin D1, and CDK4 [15]. In the presence of AZD2014, cyclin D1 and CDK4 were significantly decreased, but there were no obvious changes in the control and IR alone. In contrast, levels of cyclin B1 and CDC2, which are proteins also associated with the G2/M transition [16], treatment with IR reduced cyclin B1 and CDC2 expression, but did not alter cyclin B1 levels altered by treatment with AZD2014 (Fig 5B). Taken together, we concluded that the cytostatic effect of AZD2014 combined with ionizing radiation exhibits an inhibitory additive effect on the progression of cells through inhibits cyclin D1-CDK4 and cyclin B1-CDC2 kinase activation, leading to G1 and G2/M arrest.

### AZD2014 and radiation synergistically inhibited primary OSCC cells proliferation through inhibited AKT/mTOR and induced cell death

**Fig 6:**
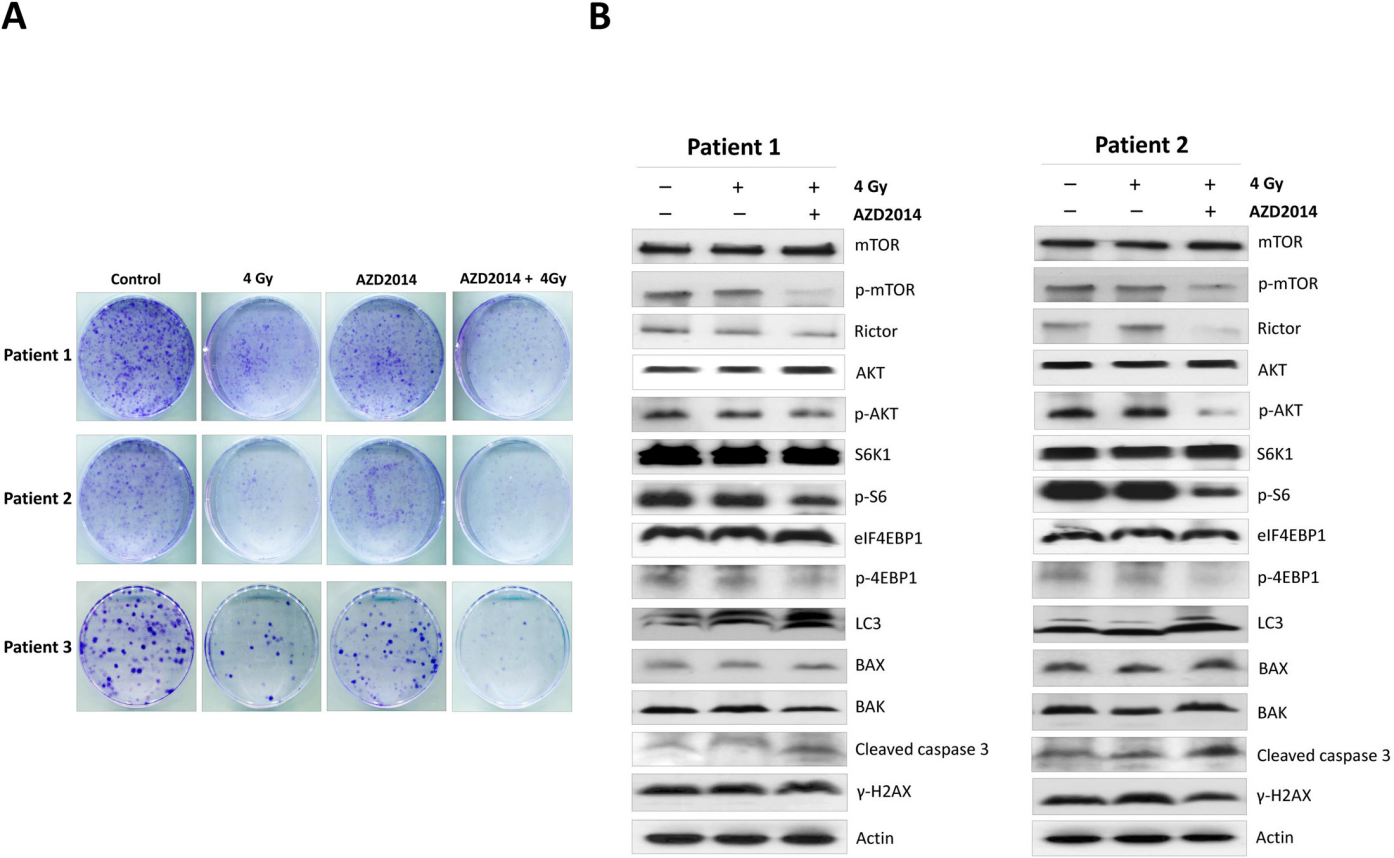
AZD2014 sensitized primary OSCC cells to radiation, which led to reduced clonogenic survival by inhibiting AKT/mTOR signaling and inducing cell death. Primary cells were established from three individual patients with OSCC, and were treated with AZD2014 alone, IR alone, or the two treatments in combination. (A) Typical image of colony growth for the different treatments are shown. (B)AKT/mTOR pathway apoptosis, autophagy, and DNA damage pathway-related proteins were analyzed by Western blotting. SD = standard deviation.

To confirm the biological effects of AZD2014, we isolated and expanded three primary tumor cells from OSCC patients and then treated the cells with AZD2014 with and without ionizing radiation. Our results indicated that combination treatment with AZD2014 and IR significantly reduced colony formation in all primary OSCC cells compared with RAD001 or RT alone (Fig 6A). To further explore the mechanisms of AKT/mTOR signaling and cell death we also found that AZD2014 treatment effectively inhibited phospho-AKT, phospho-mTOR, mTORC1 targets (p70S6K1 and 4E-BP1), and the regulatory associated protein mTORC2 (rictor). Combination treatment with AZD2014 and RT slightly increased the expression of the active caspase-3, although no significant differences in the expressions of BAX and BAK were observed. Moreover, the level of LC3 was found to be increased in combination of AZD2014 and IR, but no significant differences in γ-H2AX expression were found after AZD2014 treatment (Fig 6B). Thus, the induction of cell death and inhibition of the AKT/mTOR pathway contributed to the augmented growth-inhibitory effect induced by the combination treatment.

### AZD2014 sensitizes primary OSCC cells to radiation by inducing G1 and G2/M phase arrest

**Fig 7:**
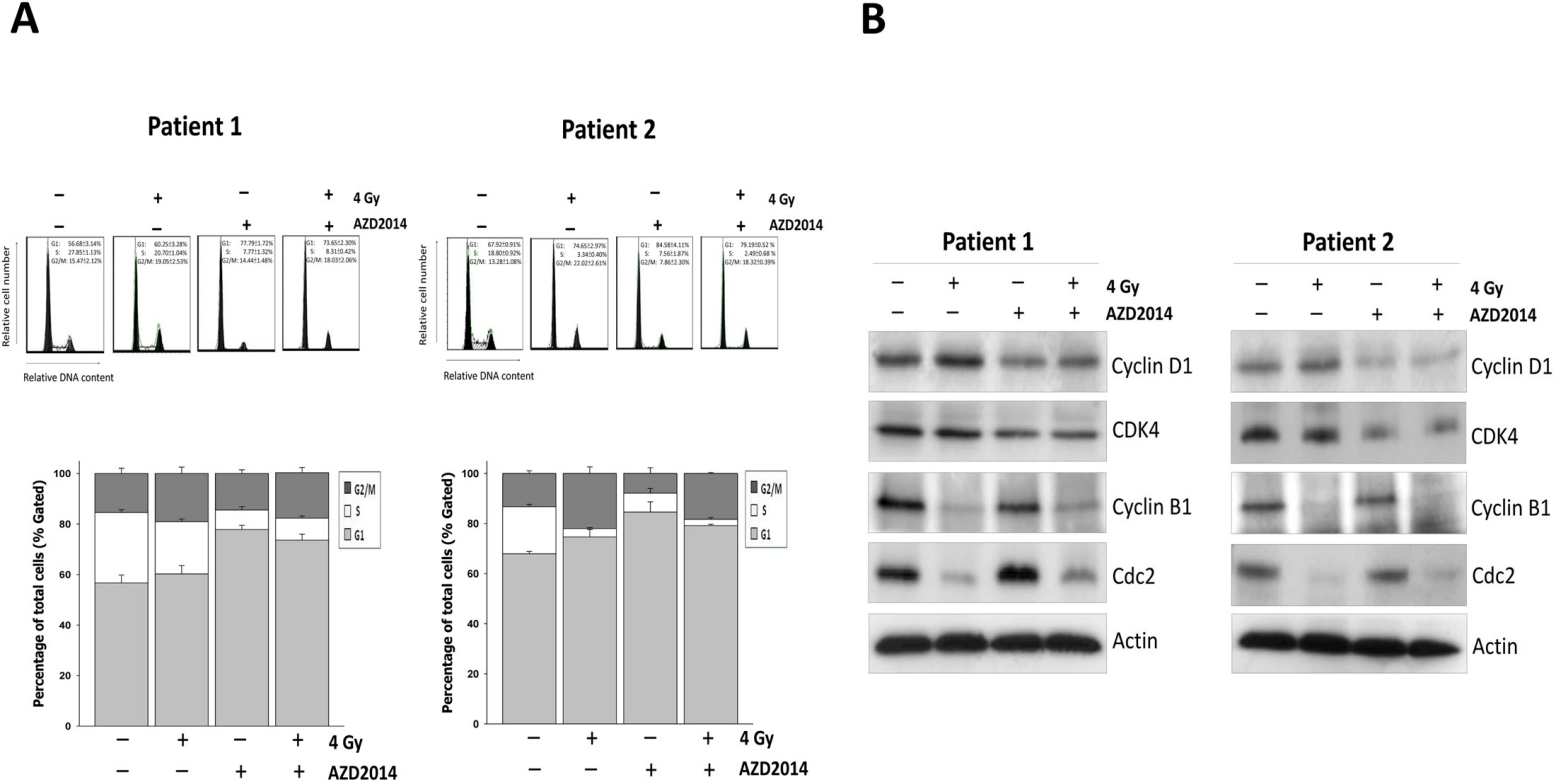
Combination treatment with AZD2014 and IR causes G1 and G2 phase cell cycle arrest in primary OSCC cells by regulating cell cycle related proteins. (A) Cell were cultured at the density of 10,000 cells in a 10 cm dish and treated with AZD2014 with or without IR for 48 hours. The cell cycle distributions were then evaluated. Data are representative of three independent experiments. (B) Expression of cell-cycle-related proteins in primary OSCC cells was determined by Western blotting.

We further determined the effect of AZD2004 on cell cycle progression in primary OSCC cells. Consistent with prior findings, AZD2014- treatment led to a more profound increase in the proportion of primary OSCC cells at G1 phase, but in the late G2/M phase of the same cell cycle being irradiated (Fig 7A). Upon exposure of cells to AZD2014 and IR an increase in the proportion of primary OSCC cells in G1 and the G2/M phase accumulation was observed with an equivalent decrease in the proportion of cells in the S phase. We also observed reduced cyclin D1 and CDK4 levels in AZD2014-treated cells, which is consistent with the compound’s ability to induce G1 arrest. Cyclin B1 and CDC2 activity were also reduced, which caused a G2/M arrest after radiation (Fig 7B).

## Discussion

The mTOR signaling pathway has emerged as an attractive therapeutic target for cancer therapy. Therefore nd inhibition of mTOR signaling as a radiosensitizing strategy in cancer cells has been investigated [11,17]. Previous studies targeting mTOR signaling in cancer have focused on allosteric inhibition of mTORC1, but not mTORC2; therefore, the anticancer activity may be attributed to incomplete inhibition of the mTOR pathway [9,18]. In the present study, we investigated the radiosensitizing effects of the dual mTORC1/mTORC2 inhibitor, AZD2014, in the OSCC cell lines and primary OSCC cells. Based on results of colony forming assays, AZD2014 significantly enhanced radiosensitization of cells by inhibiting cell proliferation.

We found that AZD2014 not only inhibited phosphorylated AKT Ser473 and phosphorylated mTOR-Ser2448, but decreased phosphorylation of the mTOR downstream targets, S6K1, and 4EBP1. Moreover, AZD2014 treatment resulted in significant inhibition of rictor, a protein component of mTORC2 expression in both cell types. mTORC2 has been considered to function mainly through activating AKT by phosphorylation at Ser473. Previous studies from the literature as well as our prior studies have reported limited anticancer efficacy of mTORC1 inhibitor due to the lack of inhibition of mTORC2 resulting in an increase in AKT activity [11,19]. Otherwise, AZD2014 treatment could cause a significant decrease in the constitutively active AKT and mTORC2 expression OSCC cells such as SCC25 cells. AZD2014 appears to exert a more potent radiosensitizing effect on OSCC cells by inhibiting AKT/mTOR signaling abrogated cell-cell induced proliferation.

The Dual m-TORC 1/2 Inhibitor AZD2014 shows a more antiproliferative potency and is involved in regulation of cell cycle[20]. As previously reported, strong G1 phase cell cycle arrest was induced by OSI-027 and AZD8055, the dual targeting of mTORC1 and mTORC2[21,22]. Several studies have found that mTORC2 regulates cell-cycle progression[23,24]. One significant finding suggests a new role for mTORC2 in the regulation of cyclin D1 stability or degradation to regulate cell growth[25]. Targeting mTORC2 has been found to suppress Cyclin D1 translation via inhibiting recruitment of Cyclin D1 mRNA to polysome in leukemia cells[26,27]. Blockage of both mTORC1 and mTORC2 has been reported to induce cell cycle arrest in G0-G1 phase and cyclin D1 and CDK4 expression levels which were significantly down-regulated in HCC cell lines via targeted inhibition of mTORC2, but not mTORC1[21].

Cyclin D1, CDK4, and CDK6 mainly act in the G1 phase. During the G1 to S cell cycle progression in response to mitogenic signals, levels of cyclin D1 increase, bind to, and activate CDK4 and CDK6 [16]. Our data indicate that inhibition of mTORC1/2 by AZD2014 resulted in G1 phase arrest, accompanied by decreased expression of cyclin D1 and CDK4. Additionally, cellular responses to radiation also induced cell cycle arrest, which has occurred through the G2/M phase delay after irradiation affecting the checkpoint to prevent cell cycle progression [28]. We have found irradiation arrests both OSCC cell lines and primary OSCC cells in the G2/M phase via disrupting cyclin B-CDC2 complex assembly, which blocks entry into mitosis. Thus, AZD2014 can be combined with radiation to significantly by enhancing the compound’s growth-inhibitory effects by causing arrest at both G1 and G2/M phases.

Radiation is a potent inducer of apoptosis, which can also be accompanied by pro-survival processes such as autophagy that contribute to an anticancer effect. In addition, several studies have demonstrated that both apoptosis and autophagy can be simultaneously induced by mTOR inhibition, to further enhance radiosensitivity in cancer cells [29,30]. We found a slight increase in LC3 protein levels in OSCC cell lines and primary OSCC cells. Although there were no differences in apoptosis-related proteins in the OSCC cell line for any of the treatments, combination treatment caused increases in caspase-3 activity in primary OSCC cells. Another study showed that increased radiosensitivity by mTOR inhibition is mediated by induction of the induced DNA damage signaling cascade [12,31]. Histone variant H2AX is a major regulator of the cellular response to DNA damage after exposure to IR [13], and has previously been shown to be associated with radiosensitization after mTOR inhibition [31]. However, we did not observe any notable differences in phosphorylation of the histone variant H2AX for any treatment.

In summary, inhibition of both mTORC1 and mTORC2 by AZD2014 was found to be more efficacious than mTORC1 inhibitor in previous our studies, the anticancer efficacy of OSCC cells. We demonstrated that AZD2014, combined with radiation, exhibits synergistic inhibition of both mTORC1 and mTORC2/AKT activity, cell cycle arrest, triggering cell death, resulting in sensitization of the OSCC cells to radiation and cell-growth inhibition.

## Supporting Information

S1 Dataset Dataset of the data used for all calculations of this article. (XLSX)
